# A network clustering based feature selection strategy for classifying autism spectrum disorder

**DOI:** 10.1186/s12920-019-0598-0

**Published:** 2019-12-30

**Authors:** Lingkai Tang, Sakib Mostafa, Bo Liao, Fang-Xiang Wu

**Affiliations:** 1 Department of Mechanical Engineering, University of Saskatchewan, Saskatoon, S7N 5A9 Canada; 2 Division of Biomedical Engineering, University of Saskatchewan, Saskatoon, S7N 5A9 Canada; 3 School of Mathematics and Statistics, Hainan Normal University, Haikou, 571158 China

**Keywords:** Autism spectrum disorder, Brain networks, Non-negative matrix factorization, Network clustering, Feature selection, Classification

## Abstract

**Background:**

Advanced non-invasive neuroimaging techniques offer new approaches to study functions and structures of human brains. Whole-brain functional networks obtained from resting state functional magnetic resonance imaging has been widely used to study brain diseases like autism spectrum disorder (ASD). Auto-classification of ASD has become an important issue. Existing classification methods for ASD are based on features extracted from the whole-brain functional networks, which may be not discriminant enough for good performance.

**Methods:**

In this study, we propose a network clustering based feature selection strategy for classifying ASD. In our proposed method, we first apply symmetric non-negative matrix factorization to divide brain networks into four modules. Then we extract features from one of four modules called default mode network (DMN) and use them to train several classifiers for ASD classification.

**Results:**

The computational experiments show that our proposed method achieves better performances than those trained with features extracted from the whole brain network.

**Conclusion:**

It is a good strategy to train the classifiers for ASD based on features from the default mode subnetwork.

## Background

Autism spectrum disorder (ASD) is a neurodevelopmental disorder characterized by repetitive social behavior, restricted interests and mental inflexibility [1]. It is estimated that 1% of global population are suffering from ASD [2]. Clinical diagnosis of ASD relies heavily on interview- or observation-based instruments [3, 4] which include interactions with clinical professionals. Thus diagnosing results could be biased by misinterpreted communication or subjective opinions of clinicians [5]. Also, diagnostic stability of such methods could be relatively low when concerning very young children [6]. Functional magnetic resonance imaging (fMRI) provides an additional approach to study brain diseases. Measuring blood oxygen level-dependent (BOLD) signals, fMRI is a non-invasive scanning technique showing fluctuations of functional activities of a whole brain. As the center of nervous system, a human brain can be considered as a complex system where different regions have different functions and regions cooperate with each other to perform certain cognitive functions. Correlation of BOLD signals among brain regions indicate underlying functional interactions.Biswal et al. [7] demonstrates that even though brain is at the resting state, regions that frequently interact with each other at the normal state can still have strong correlations. Thus, the resting-state fMRI (rs-fMRI) can provide an intrinsic functional mapping and has been widely used in studying the functional organization of brains.

Considering the massive functional correlations between brain regions, we can naturally view a brain as a network, where regions are vertices and functional correlations are edges [8]. Previous studies have shown that functional brain networks (FBN) have certain small-world properties, such as high clustering coefficient or short characteristic path length [9]. Vertices in such networks are prone to form modules. This agrees with our understanding that brains are modular systems where different brain parts have separated functions. Implementations of network clustering methods have successfully identified modules in different contexts. Power et al. [10] identify modules using rs-fMRI images and mapped modules to cognitive function. Crossley et al. [11] use task-evoked fMRI images and link their identified modules to 4 types of behaviours. In addition, alterations in modules with aging or cognitive status are also studied [12]. These studies successfully map the functional organization of brain to FBNs.

Previous researches have revealed associations between alterations in rs-fMRI images or derived FBNs and pathology of ASD. A majority of studies have discovered that children with ASD have increased total brain volumes [13]. A decrease of global network efficiency is also reported via studying FBNs [14]. Recent researches have also successfully implemented machine learning algorithms in analyzing FBNs of ASD subjects, performing automated classification and offering complementary methods for clinical diagnosis. Several classifiers and forms of features have been implemented to diagnose ASD. Plitt et al. [15] use functional correlations as features to train different classifiers such as linear support vector machine (SVM), random forest (RF), linear discriminant analysis (LDA), Lasso-regularized logistic regression (LRLR) and k nearest neighbors (kNN). In particular, classifiers are trained with correlation values in FBNs. Chen et al. [16] use two feature selection strategies named particle swarm optimization (PSO) and recursive feature elimination (RFE), combined with SVM classifier and obtains accuracies about 80% and 100%, respectively, on training data but much less on testing data. PSO iteratively optimizes the positions of particles according to certain cost function measuring the quality [17]. For feature selection purpose, the position of a particle is represented by a binary vector whose components indicate whether a feature participates the training process. The cost function usually measures the performance of the classifier [18]. RFE ranks all the features and recursively eliminate bottom-ranked ones [19]. Price et al. [20] use dynamic functional correlations obtained from multiple networks from large time scales and Tolan et al. [21] add centrality-based indices to the collection of features. Developments in deep learning also inspire new methods for ASD diagnosis. Guo et al. [22] use deep neural networks in feature selection and classification, achieving accuracy over 80%. Autoencoders, as another form of artificial neural network, is implemented for the identification of ASD [23] with accuracy about 70%.

Aforementioned studies about classification extract features from the whole brain network. Therefore, the dimension of feature vectors could be relatively large and thus not very discriminant. In addition, high dimensional feature vectors could cause overfitting issue and increase computational complexity. In this study, we introduce a new strategy to extract the features for classifiers from a network module. In particular we present the joint symmetrical non-negative matrix factorization (JSNMF) to cluster FBNs into several modules. Non-negative matrix factorization (NMF) is an unsupervised machine learning method. NMF has been widely used in identifying communities in complex networks such as social networks [24] or biomolecular networks [25]. Ordinary NMF methods factorize one matrix a time, but real-world datasets may contain multiple views, or attributes which complement each other. Liu et al. [26] introduces a multi-view clustering algorithm by formulating a joint cost function meanwhile keeping clustering results meaningful. Ou [27] and Zong [28] add regularization terms in cost functions to preserve local geometrical structures. Such joint NMF methods are also successfully implemented on biological datasets. Zhang et al. [29, 30] propose methods for clustering ovarian cancer samples with several types of data including gene expression data, microRNA data, etc. Breast cancer samples are also studied with similar types of data [31]. For network clustering propose, Zhang et al. [32] identify communities from social networks at different time points. For brain networks, we can also regard each individual FBN as a view since it represents a different organization of connectivity that human brain may have. Although individual FBNs vary at local connections, all subjects may have similar modular structure, considering that cortical regions of different subjects are similar if they share one cognitive function. Our proposed JSNMF method solves a regular symmetrical NMF cost function but in a joint form to obtain a consensus that contains lower dimensional features valid for all individual FBNs.

**Fig. 1:**
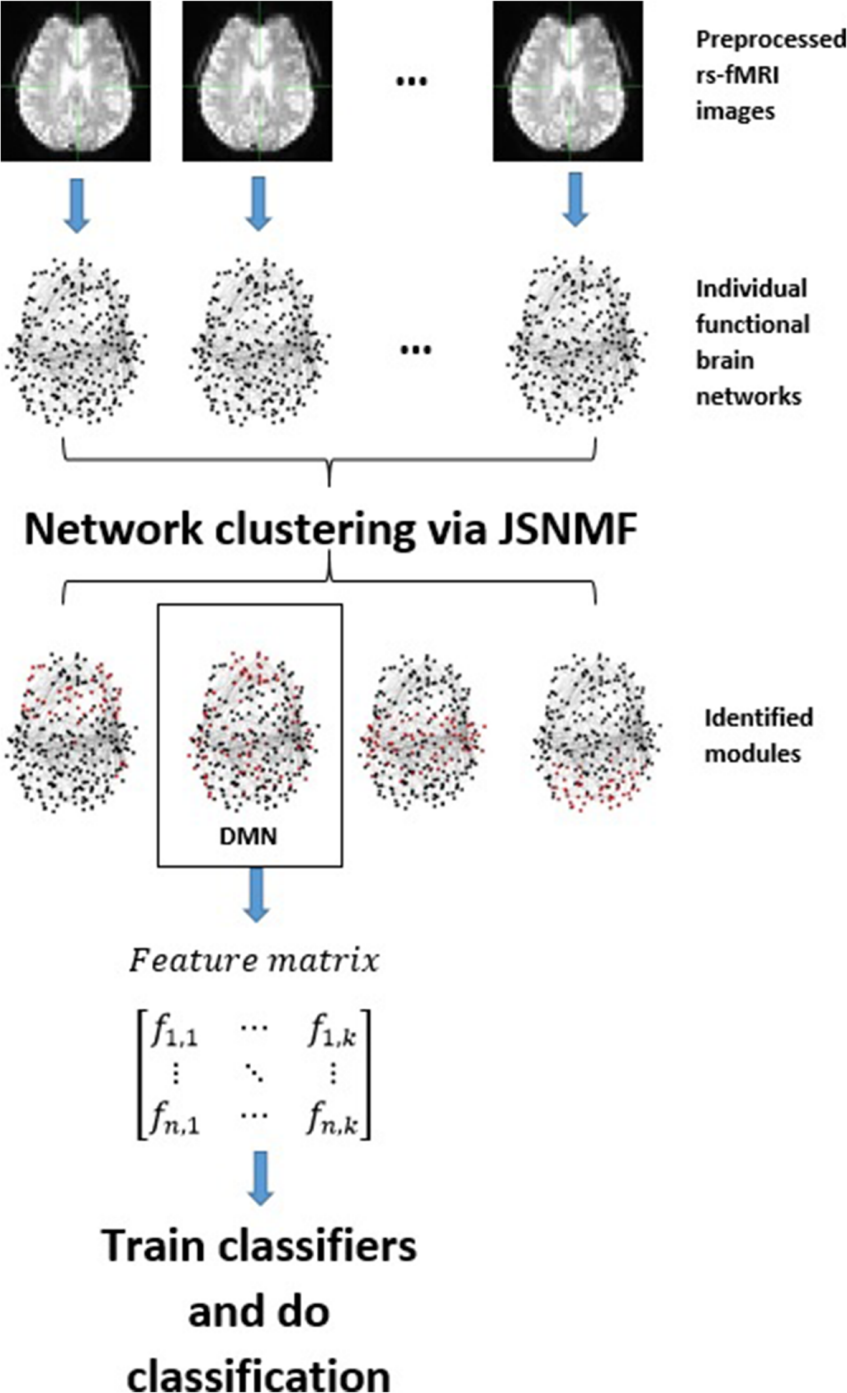
A flowchart showing the whole pipeline, including building FBNs, module identification, feature extraction and classification

We extract features from one module and train several commonly used classifiers [15, 16, 22]. The flowchart showing our whole pipeline is given in Fig. 1. We compare classification performance between features extracted from one module and the whole brain. The results show that the performances of classifiers trained with the features from a module are better than those trained with features from a whole network.

## Methods

### Dataset

#### Acquisition and preprocessing of rs-fMRI data

All rs-fMRI data were acquired at UCLA on a Siemens 3 T Trio scanner. Configuration of the scanning can be found in [33] and image data can be obtained from ABIDE dataset [34]. The rs-fMRI images are preprocessed with FMRIB Software Library (FSL) [35] and Analysis of Functional NeuroImages (AFNI) [36], by following a pipeline introduced in [33]. Brain-only images are extracted from surrounding skulls and tissues with AFNI. Functional volumes along time are motion corrected with FSL MCFLIRT [37] and registered to a mean volume using a normalized correlation cost function and sinc interpolation. 6 parameters of rigid body movement are calculated for each volume and if the average displacement over all voxels between two consecutive volumes is above 2.5 mm, we consider this subject as a outlier and stop any further processing. 9 nuisance regressors, including 6 rigid body movement parameters and average BOLD signals of white matter, cerebrospinal fluid and whole-brain, are regressed out of all volumes. White matter and cerebrospinal fluid are segmented with FSL FAST [38].Images are applied a Gaussian kernel with full width at half maximum of 5 mm for spatial smoothing and filtered with a band-pass filter of Hz to reduce influence of heart beat and breath. Images of all subjects are registered to MNI 152 standard space using FSL FLIRT with affine transformation and mutual information cost function.

#### Constructing FBNS

A recent study introduced a whole-brain parcellation based on meta-analysis of fMRI, yielding 264 ROIs in MNI 152 standard space. Compared with traditional anatomical atlases, this parcellation avoids large ROIs containing several different functional regions, so that BOLD signals representing different functions will not be mixed. Then we calculated the Pearson correlation coefficients of average signals between every pari of ROIs to obtain a 264×264 adjacency matrix for each subject.

### JSNMF

Given a multiview dataset *A*={*A*^(1)^… *A*^(*n*)^}, where *A*^(*v*)^ in our case is a 264×264 FBN adjacencey matrix of a subject and *n*=37. JSNMF solves an optimization problem by minimizing the following objective function

1 $$\begin{array}{@{}rcl@{}}  O_{JSNMF}=\sum_{v=1}^{n}{\left\| A^{(v)}- HS^{(v)}H^{T} \right\|}^{2}_{F} + 4\alpha \sum_{k=1}^{K}{\left|h_{k}\right|}_{1} \\ s.t.\quad H\ge 0 \quad and \quad S^{(v)} \ge 0 \quad for \quad v=1, \ldots, n \end{array} $$

where ∥∗∥_*F*_ represents the Frobenius norm of a matrix, *h*_*k*_ is the *k*-th column of matrix *H*∈*R*^*N*×*K*^ where *K* is the number of modules desired, and ∥∗∥_1_ represents the *L*_1_−*norm* of a vector to make it sparse, *α* is a positive regularization factor.

To minimize *O*_*JSNMF*_, we can introduce Lagrangian multiplier *Λ*∈*R*^*N*×*K*^ and rewrite equation 1 as

2 $$  O_{L}\,=\,\sum_{v=1}^{n}{\left\| A^{(v)}\,-\, HS^{(v)}H^{T} \right\|}^{2}_{F} \!+ 4\alpha \sum_{k=1}^{K}{\left|h_{k}\right|}_{1} \,+\, Tr(H^{T}\Lambda),  $$

where *TR*(∗) is the trace of a matrix. The partial derivatives of equation 2 with respect of *S*^(*v*)^ and *H* are respectively as follows

3 $$\begin{array}{*{20}l} &\frac{\partial O_{L}}{\partial S^{(v)}} = -2H^{T}A^{(v)}H + 2H^{T}HS^{(v)}H^{T}H \end{array} $$

4 $$\begin{array}{*{20}l} &\frac{\partial O_{L}}{\partial H} \,=\, \sum_{v=1}^{n}\left(-4A^{(v)}HS^{(v)} + 4HS^{(v)}H^{T}HS^{(v)}\right)\\&\quad\quad\quad + \Lambda + 4\alpha E, \end{array} $$

where *E*=1^*N*×*K*^. Solving $\frac {\partial O_{L}}{\partial S^{(v)}} = 0$, we get

5 $$\begin{array}{@{}rcl@{}}  S^{(v)}=(H^{T}H)^{-1}H^{T}A^{(v)}H(H^{T}H)^{-1}. \end{array} $$

According to the Karush-Kuhn-Tucker (KKT) conditions [39], we obtain

6 $$  {}H \odot \left(\sum_{v=1}^{n}\left(-4A^{(v)}HS^{(v)} + 4HS^{(v)}H^{T}HS^{(v)}\right) + \alpha E\right)=0,  $$

where ⊙ represents Hadamard production. Therefore, we can obtian the following update rules

7 $$ {\begin{aligned} H \leftarrow H \odot \left(\sum_{v=1}^{n}(A^{(v)}HS^{(v)}) \oslash \left(\sum_{v=1}^{n}(HS^{(v)}H^{T}HS^{(v)})\right)+\alpha E\right), \end{aligned}}  $$

where ⊘ represents element-wise division of matrices. The non-negative matrix *H* *and* *S*^(*v*)^ is randomly initialized and updated by following equations 6 and 8 until converged. Guarantee of convergence can be easily proved as in the literature [40–42].

The matrix *H* is the cluster indicator and it is normalized so that the maximum value of each column equals to 1 to balance the sizes of modules. A vertex is assigned to a module where the element value of its corresponding row reaches the maximum, i.e., vertex *i* belongs to module *k* if the *H*_*i*,*k*_ is the largest one in *i*-th row of normalized *H*.

### Evaluation indices of clustering performance

We use three indices to measure the quality of modules: modularity, conductance and coverage. Let *A* be a FBN adjacency matrix and (*M*_1_,…,*M*_*K*_) represent the *K* modules identified. Let $V_{k} = \sum _{i \in M_{k}} \sum _{j=1}^{N}A_{i,j}$ and $W_{k} = \sum _{i \in M_{k}, j \in M_{k}}A_{i,j}$ for *k*=1,…,*K*.

Modularity measures the quality of modules of higher intra-community connections than the expected random connections of the vertices with probabilities proportional to their degrees and is calculated as follows [43]:

8 $$\begin{array}{@{}rcl@{}}  Mod(M_{1}, \ldots,M_{K}) = \sum_{k=1}^{K}(W_{k}-V_{k}^{2}). \end{array} $$

The higher modularity is, the better the clustering method is.

Conductance measures the possibility of a one-step random walk entering or leaving a module [43] and it is defined as

9 $$\begin{array}{@{}rcl@{}}  Con(M_{1}, \ldots,M_{K}) = \frac{1}{K} \sum_{k=1}^{K} \frac{W_{k}}{V_{k}}. \end{array} $$

The more difficult a random walk leaving or entering a module, the stronger connectivity is inside the module, which means the modules is more compact.

Coverage measures the fraction of intra-module connections over all connections [43] and is calculated as

10 $$\begin{array}{@{}rcl@{}}  Cov(M_{1}, \ldots,M_{K})=\sum_{k=1}^{K} W_{k}. \end{array} $$

The denser a module is, the higher the coverage value is.

### Measuring performances of classifiers

In this study, we measure the performances of classifiers by drawing ROC curves and calculating AUCs. The ROC curves are plotted with the true positive rate (TPR) against the false positive rate (FPR) over a series of classification thresholds. TPR, also called sensitivity, measures the proportion of positive samples that are correctly predicted over all actual positive samples, while FPR measures the proportion of samples wrongly predicted as positive over all actual negative ones. Thus, TPR and FPR can be respectively defined as

11 $$\begin{array}{@{}rcl@{}}  TPR = \frac{TP}{P} \end{array} $$

12 $$\begin{array}{@{}rcl@{}} FPR = \frac{FP}{N}, \end{array} $$

where TP and FP are the numbers of samples correctly or wrongly predicted as positive, respectively, and P and N are numbers of actual positive or negative samples, respectively. In medical diagnosis, TPR is the ability of a test to correctly identify diseased samples, while FPR measures the rate of healthy samples misdiagnosed with the disease.

To draw the ROC curves, we need to set up a series of classification thresholds. In classification, the thresholds are designed for the output value of a classifier to determine which class a sample belongs to. ROC curves depicts the performance of classifiers under different thresholds and help choose the threshold yielding best performance. The ROC curve of a perfect classifier should reach point (0,1) in ROC space, representing 100% TPR and 0% FPR.

AUC is the area under the ROC curve and measures the general performance of a classifier. If the AUC is large, it means the ROC curve is close to point (0,1), or at least it partially has high TPR or low FPR. AUC is defined as

13 $$\begin{array}{@{}rcl@{}}  AUC = \int_{0}^{1} TPR \quad dFPR. \end{array} $$

In practice, AUC is approximated by the trapezoidal numerical integration.

## Results and discussions

### Clustering of FBNS

We first implement our network clustering algorithm, JSNMF, and run it on a dataset collected from UCLA Multimodal Connectivity Database [44]. This dataset contains 42 individual functional networks of subjects from ASD group and 37 individual functional networks from typically developed (TD) group. Each of these functional networks is a weighted network consisting of 264 nodes (regions of interest, ROIs) and the edges are weighted by the Pearson correlation coefficient of the time series BOLD of two ROIs. AS in the literature [33], all negative weights are firstly removed. To filter out the edges with small weights which are possibly generated by noise [9] while keeping all individual networks connected, the edges with their weights less than a threshold of 0.35 are further removed. Note that the adjacency matrices of resultant networks are symmetric and nonnegative. At this stage, for clustering purpose, we only use TD networks to guarantee the quality of the identified modules, since ASD could alter the modular organization of brains [45].

We run the JSNMF algorithm in MATLAB R2013a with different settings of parameters: *K*, the number of modules and *α*, the regularization factor. The performance of the algorithm is measured by modularity, conductance and coverage [43] and these 3 indices are calculated on an average network and each individual network. The performance of our method JSNMF are shown in Tables 1 and 2, where we fix one parameter and change the other one. For both tables, top half are the average values of three indices over 37 individual networks, while the bottom half are the values of those three indices on the average network. We calculate the indices on individual networks because we believe the clustering results should be valid for all subjects, even though their FBNs are not quite similar. As shown in Table 1, modularity reaches the maximum when *K*=4 and *α* fixed to 1 while Table 2 shows that all three indices is maximized when *α*=1. Therefore, we set the parameters as *K*=4 and *α*=1.

**Table 1:** Performance of JSNMF with different settings of *K* when *α* fixed to 1

*K*	Modularity
Average over all individual networks	
2	0.2696
3	0.3290
4	0.3454
5	0.3245
6	0.3056
7	0.2901
8	0.2754
9	0.2632
10	0.2532
Average network	
2	0.2727
3	0.3325
4	0.3473
5	0.3258
6	0.3076
7	0.2925
8	0.2760
9	0.2635
10	0.2528

**Table 2:** Performance of JSNMF with different settings of *α* when *K* fixed to 4

*α*	Modularity	Conductance	Coverage
Average over all individual networks			
0	0.3449	0.5905	0.6101
0.1	0.3451	0.5905	0.6105
1	0.3454	0.5909	0.6105
10	0.3453	0.5908	0.6103
100	0.3452	0.5909	0.6101
Average network			
0	0.3470	0.5930	0.6084
0.1	0.3473	0.5929	0.6084
1	0.3475	0.5933	0.6088
10	0.3474	0.5933	0.6086
100	0.3473	0.5933	0.6084

To demonstrate our method has better performance, we compare it with two other methods: multiclass spectral clustering (MSC) [46] and co-regularized multi-view spectral clustering (CMSC) [47]. We consider them as competing methods because all three methods are based on matrix factorization and dimension reduction. The two competing methods are also implemented in MATLAB R2013a and different parameter settings are tested to find their best performance. Table 3 collects the results of all three methods. From Table 3, we can see that our method perform the best in terms of modularity while ranking at the second or the third in terms of coverage or conductance. However, it is believed that the modularity is the most powerful index to measure the quality of the network clustering [48].

**Table 3:** Comparison of performances of different methods

Methods	Modularity	Conductance	Coverage
Average over all individual networks			
MSC	0.3441	0.5937	0.6040
CMSC	0.3451	0.5920	0.6119
JSNMF	0.3454	0.5909	0.6105
Average network			
MSC	0.3454	0.5953	0.6016
CMSC	0.3469	0.5939	0.6099
JSNMF	0.3475	0.5933	0.6088

Since our algorithm is randomly initialized, the resultant modules may be different in each run. Using the adjusted rand index (ARI) [49], we calculate similarities between modules of any two runs. We find that the lowest ARI value among all pairs is 0.91 which is pretty high, indicating high similarities among runs and the robustness of our algorithm. Therefore, we choose the result from one run that is most similar to results of all other runs for the following stages.

### Classification of ASD with default mode network features

**Fig. 2:**
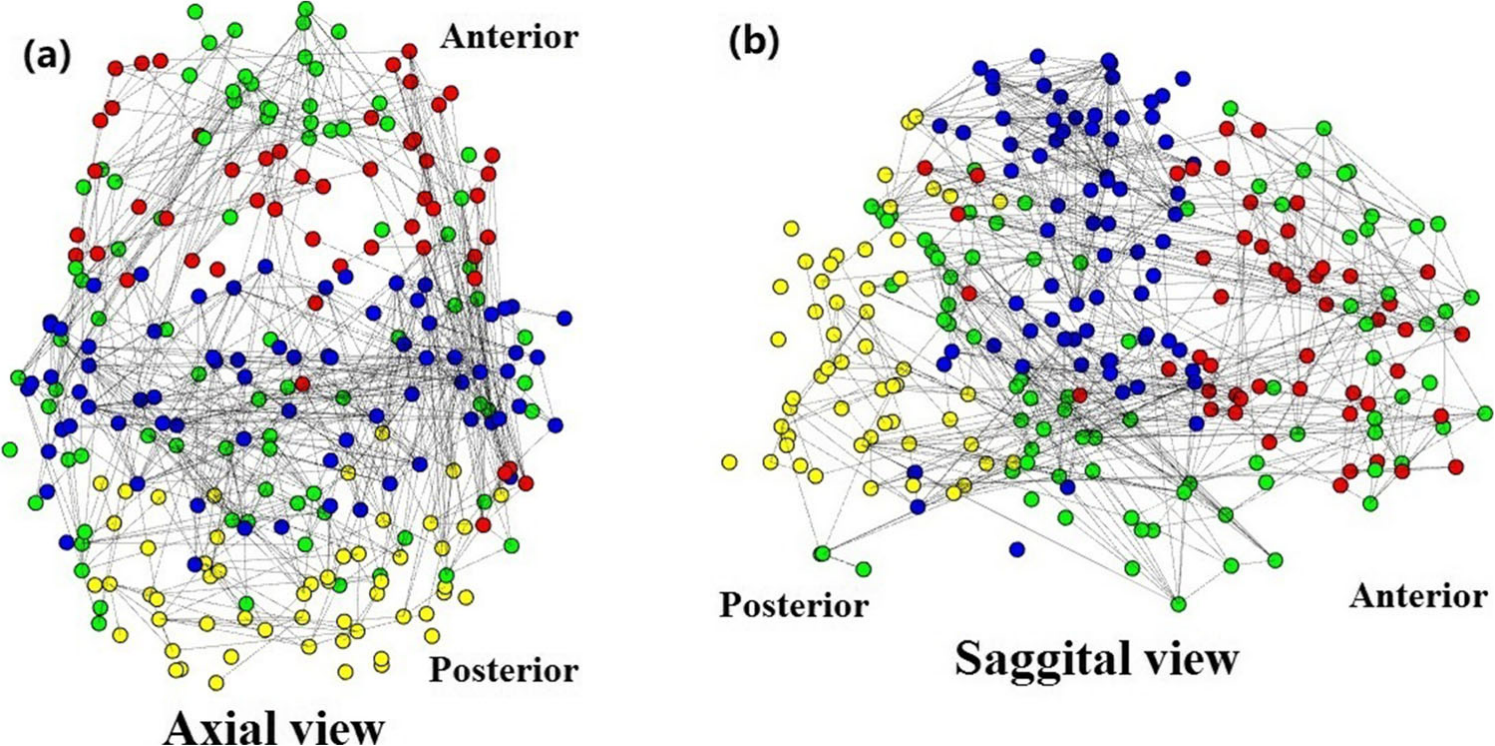
Three dimensional views show the average FBN. The vertices are aligned with coordinates in MNI 152 standard space. Only correlations higher than 0.8 are displayed. Vertices in DMN are shown in green

Default mode network (DMN) which is a brain module identified in several researches based on fMRI images or FBNs [50] and considered to be responsible for many cognitive functions [51]. Figure 2 shows the 4 different color-coded modules identified with our JSNMF. The green module is corresponding to the DMN, which mainly expands in middle and inferior temporal gyrus, cigulate gyrus, hippocampal gyrus, frontal gyrus and their surrounding regions and contains several previously identified core regions of DMN including Medial prefrontal cortex, posterior cingulate cortex and hippocampal formation [50]. In addition, studies [45] have reported the participation of temporal lobule in DMN for certain functions. Medial prefrontal cortex shows increasing volumes in ASD subjects [52, 53]. Average FBN and integration of DMN are also reported to decrease with severity of ASD [54–56]. Therefore, we extract features from this green module in this study.

Specifically, the Pearson correlation coefficients are extracted from all pairs of ROIs in DMN and whole-brain networks for each of all ASD and TD subjects, respectively and are arranged as their feature vectors to train several classifiers. We compare the performances of classifiers trained with features from DMN and whole-brain networks. The classifiers include linear SVM, particle swarm optimization SVM (PSOSVM) [18], recursive feature elimination SVM (RFESVM) [19], RF, LDA, LRLR and kNN. We choose these classifiers because they were previously trained with the features defined by Pearson correlation coefficients from the whole-brain network[15, 16]. In addition, in those studies the networks are built following a similar pipeline as in this study. All classifiers are implemented in MATLAB Machine Learning Toolbox and Scikit-learn in Python and are evaluated with the leave-one-out cross validation.

**Fig. 3:**
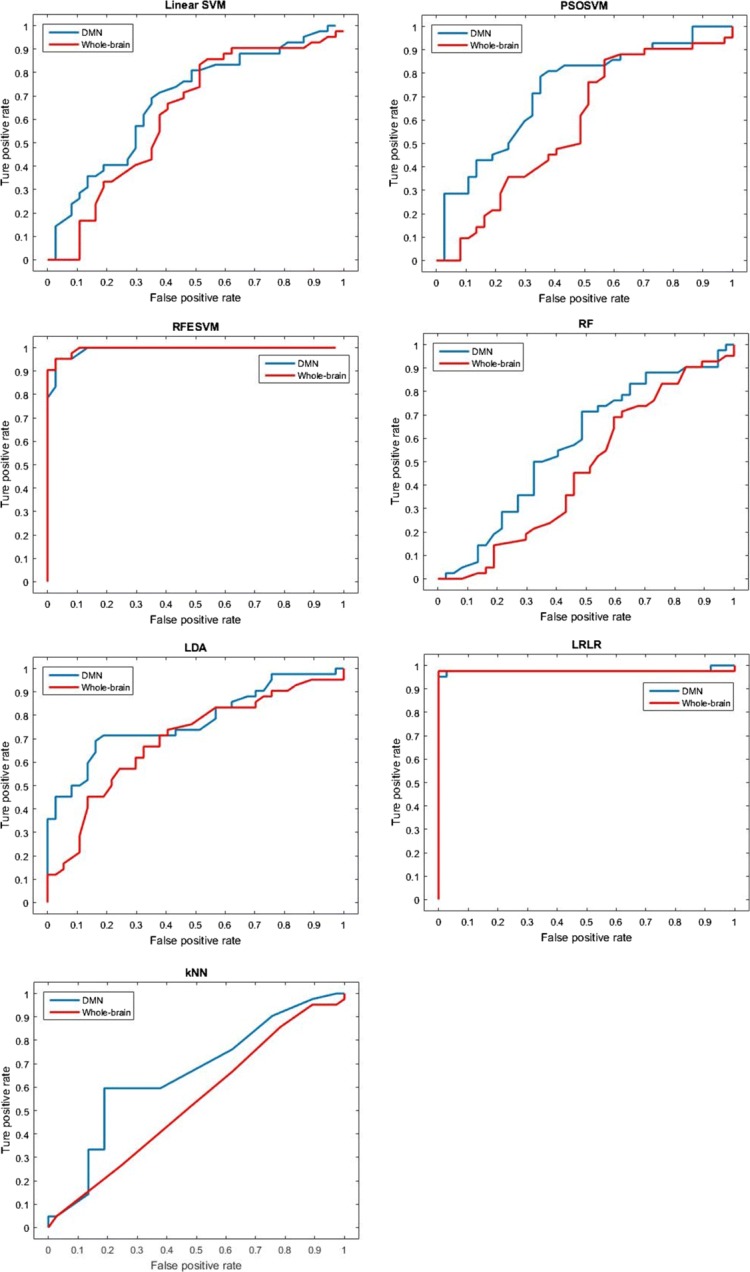
The ROC curves of classifiers trained with DMN features and whole-brain features. For SVM based classifiers, the classifying thresholds range from the smallest values the test data can reach, to the largest ones. And for other classifiers, the thresholds range from 0 to 1

We draw the receiver operating characteristic (ROC) curves and calculate the area under curves (AUC) to measure the performance of each classifier. Figure 3 shows that for all classifiers, except for RFESVM, DMN features yield higher AUC than whole-brain features. For RFESVM, the performances of two feature sets are basically the same and they are both relatively high. We can also see that DMN features outperform whole-brain features especially at low false positive rate (FPR). Considering the application in clinical diagnosis, which requires the low misdiagnosis rate, DMN features have more potential for clinical trials. In addition, we can see from Table 4 that both PSOSVM and RFESVM classifiers with extra feature selection stages have higher AUCs than most of the others, indicating the potential of classification performance can be further improved with the feature selection strategy.

**Table 4:** AUCs of classifiers trained with DMN and whole-brain features

Classifier	Linear SVM	PSOSVM	RFESVM	RF	LDA	LRLR	kNN
DNM features	0.6264	0.7215	0.9640	0.5769	0.7754	0.9775	0.6541
Whole-brain features	0.5171	0.5822	0.9675	0.4678	0.6943	0.9762	0.5347

Compared with previously developed classification methods [22, 57, 58], our strategy is easier to implement because modular information is highly accessible. In this study, we develop a new clustering algorithm to find functional modules but it is possible to use other cortex parcellation schemes [10, 11, 59], including anatomical parcellations which has been integrated in many brain image analysis tools. Some previously used feature selection strategies are quite complicated, especially when neural networks are involved [22, 57, 58, 60]. Our strategy takes less time and can also achieve high performance. In addition, our strategy can be integrated with other classification methods. Previous methods can be easily implemented on modular features, and since number of features is smaller, it would take less time to train the classifiers or select more discriminant features with other strategies.

## Conclusion

In this study, we have proposed a new strategy to select discriminant features for the classification of ASD. The experiment results show that classifiers trained with features extracted from a single brain module named DMN generally perform better than those trained with features extracted from a corresponding whole-brain network. In addition, this strategy can greatly reduce the numbers of features, which not only yield less computational complexity and shorter training time, but also potentially avoid the overfitting problem.

As indicated with PSOSVM and RFESVM classifiers, further feature selection could improve the performance of classifiers. Therefore, one direction of our future work is to effectively incorporate our proposed strategy in this study with other feature selection method to further improve the performance of classifiers. Beside the functional MRIs, there are also other brain imaging modalities such DTI and CT. Another direction of out future work is to integrate multi-modalities of brain imaging to study the classification of ASD.

## Data Availability

The dataset analyzed during the current study are available in UCLA Multimodal Connectivity Database. http://umcd.humanconnectomeproject.org

## Abbreviations

AFNI: Analysis of functional neuroimages
ARI: Adjusted rand index
ASD: Autism spectrum disorder
AUC: Area under curve
BOLD: Blood oxygen level-dependent signals
CMSC: Co-regularized multi-view spectral clustering
DMN: Default mode network
FBN: Functional brain networks
fMRI: Functional magnetic resonance imaging
FPR: False positive rate
FSL: FMRIB software library
JSNMF: Joint symmetrical non-negative matrix factorization
KKT: Karush-Kuhn-Tucker
kNN: k nearest neighbors
LDA: Linear discriminant analysis
LRLR: Lasso-regularized logistic regression
MSC: Multiclass spectral clustering
NMF: Non-negative matrix factorization
PSO: Particle swarm optimization
PSOSVM: Particle swarm optimization support vector machine
RF: Random forest
RFE: Recursive feature elimination
RFESVM: Recursive feature elimination support vector machine
ROC: Receiver operating characteristics
ROI: Region of interest
rs-fMRI: Resting state functional magnetic resonance imaging
SVM: Support vector machine
TD: Typically developed
TPR: True positive rate
UCLA: University of California, Los Angeles
